# The irtQ R package: a user-friendly tool for item response theory-based test data analysis and calibration

**DOI:** 10.3352/jeehp.2024.21.23

**Published:** 2024-09-12

**Authors:** Hwanggyu Lim, Kyungseok Kang

**Affiliations:** College of Education, Inha University, Incheon, Korea; Hallym University, Korea

**Keywords:** Algorithms, Calibration, Computerized adaptive testing, Likelihood functions, Psychometrics

## Abstract

Computerized adaptive testing (CAT) has become a widely adopted test design for high-stakes licensing and certification exams, particularly in the health professions in the United States, due to its ability to tailor test difficulty in real time, reducing testing time while providing precise ability estimates. A key component of CAT is item response theory (IRT), which facilitates the dynamic selection of items based on examinees' ability levels during a test. Accurate estimation of item and ability parameters is essential for successful CAT implementation, necessitating convenient and reliable software to ensure precise parameter estimation. This paper introduces the irtQ R package (http://CRAN.R-project.org/), which simplifies IRT-based analysis and item calibration under unidimensional IRT models. While it does not directly simulate CAT, it provides essential tools to support CAT development, including parameter estimation using marginal maximum likelihood estimation via the expectation-maximization algorithm, pretest item calibration through fixed item parameter calibration and fixed ability parameter calibration methods, and examinee ability estimation. The package also enables users to compute item and test characteristic curves and information functions necessary for evaluating the psychometric properties of a test. This paper illustrates the key features of the irtQ package through examples using simulated datasets, demonstrating its utility in IRT applications such as test data analysis and ability scoring. By providing a user-friendly environment for IRT analysis, irtQ significantly enhances the capacity for efficient adaptive testing research and operations. Finally, the paper highlights additional core functionalities of irtQ, emphasizing its broader applicability to the development and operation of IRT-based assessments.

## Graphical abstract

[Fig f5-jeehp-21-23]

## Introduction

### Background

Computerized adaptive testing (CAT) has become a widely adopted method for high-stakes licensing and certification exams in the United States, particularly in the health professions (e.g., National Council Licensure Examination, North American Pharmacist Licensure Examination). Its ability to tailor test difficulty in real-time, reducing testing time while providing precise ability estimates, has attracted increasing attention from health-related fields worldwide. In Korea, for example, discussions have emerged around using CAT for national medical licensing exams, with proposals focusing on its potential to enhance both efficiency and fairness in testing [1-3].

In CAT, item response theory (IRT) plays a critical role in many of its key functionalities, although some CAT implementations may use alternative approaches, such as tree-based CAT [4]. For example, IRT is essential for updating an examinee’s ability estimate throughout the test, based on their responses and the characteristics of the test items, such as difficulty and discrimination. This dynamic estimation is central to the adaptive nature of CAT, where optimal items are selected from an item pool using criteria like maximum Fisher information, which is computed from IRT-based item parameters. It is crucial to estimate item parameters accurately during the initial development phase of the item bank to ensure the validity and reliability of CAT. Furthermore, as the CAT evolves, new pretest items must be calibrated under the IRT model to ensure they align with the scale of item parameters in the bank before being integrated into the operational pool. This step is vital for maintaining test security and ensuring the exam continues to measure abilities accurately.

A critical step in applying IRT in CAT is accurately estimating item parameters, such as difficulty and discrimination, which define the chosen IRT models and inform examinees’ ability estimates. The effectiveness of CAT depends on the precision of these estimates, as they directly impact item selection and the accuracy of ability measurement. Thus, the successful implementation of IRT in CAT requires robust statistical methods and reliable software to ensure precise parameter estimation.

Therefore, this study aims to introduce the R software package, irtQ, a user-friendly tool available on CRAN (http://CRAN.R-project.org/) for efficiently conducting IRT modeling [5]. The irtQ package is designed to simplify the process of analyzing test data using unidimensional IRT models, providing researchers and practitioners with a convenient solution to enhance the accuracy and efficiency of testing procedures. While irtQ does not directly simulate CAT, it offers essential resources for supporting CAT-related research and operations. These features include fitting unidimensional IRT models to test data using marginal maximum likelihood estimation via the expectation-maximization algorithm (MMLE-EM) [6], calibrating pretest items through fixed item parameter calibration (FIPC) [7], and implementing fixed ability parameter calibration (FAPC) [8]. The package further allows users to estimate examinees’ latent ability parameters and evaluate the fit of IRT models at the item level (e.g., using *χ*^2^ fit statistics), offering a range of additional IRT-related functions.

### Objectives

In this paper, we explore the key functions of irtQ that are particularly beneficial for developing and operating CATs, illustrating their application through practical examples. First, we reviewed the dichotomous IRT models implemented in the irtQ package and described the item metadata format, which serves as both input and output for IRT analyses. Next, we illustrated how to use the core functions of irtQ to analyze test data with simulated response datasets. Finally, a summary and the limitations were presented.

## Fundamentals of using irtQ for dichotomous IRT models

This section focuses exclusively on the dichotomous IRT models available in the irtQ package. These models are designed for items scored as either correct or incorrect and include the 1-parameter logistic model (1PLM), 2-parameter logistic model (2PLM), and 3-parameter logistic model (3PLM). While irtQ also supports polytomous models (e.g., graded response model, generalized partial credit model), we emphasize dichotomous models, as they are widely used in adaptive testing and traditional assessments. For the IRT 1-3PLMs, the probability that an examinee with ability *θ* answers an item correctly is given by:


(1) $$P\left(Y=1\text{|}\theta \right)=g+\frac{1-g}{1+\text{exp}\left[-Da\left(\theta -b\right)\right]}$$

where *a* is the item discrimination parameter, *b* is the item difficulty parameter, and *g* is the guessing parameter. The scaling constant *D* is typically set to 1.702 to align the logistic function with the normal ogive curve. In the 1PLM, *a* is constrained to be equal across all items (typically fixed to 1), while in the 2PLM, *a* varies for each item. In both 1PLM and 2PLM, the guessing parameter *g* is set to 0. The 3PLM introduces *g* to account for random guessing for examinees with extremely low ability levels.

### Item metadata

In the irtQ package, item metadata, a specialized data frame, serves as an essential component of both input or output data for various IRT analyses, such as item calibration, ability estimation, and the computation of item and test information functions or characteristic curves. The metadata contains essential details for each item in a test, including the item’s identifier (ID), the number of score categories, the IRT model applied, and the associated item parameters. The supported IRT models for dichotomous items are 1PLM, 2PLM, 3PLM, and dichotomous response model (DRM), where DRM encompasses all dichotomous IRT models. From the fourth column onward, item parameters are provided. For dichotomous items, the fourth, fifth, and sixth columns represent the item’s discrimination, difficulty, and guessing parameters, respectively. If an item is modeled using the 1PLM or 2PLM, the guessing parameter (sixth column) should be filled with NA for that item. An example of item metadata for a test with 5 items is shown in Table 1.

The shape_df() function in the irtQ package simplifies the creation of item metadata by allowing users to define the item parameters and automatically structure them into a usable format. Below is an example of creating item metadata for 5 dichotomous items, as presented in Table 1.

R> library(irtQ)

R> x <- **shape_df**(par.drm = **list**(

R> a = **c**(1, 1.2, 0.5,1.7, 0.7),

R> b = **c**(0.2, 2.3, -0.4, -2.5, 0.9),

R> g = **c**(NA, NA, 0.2, 0.1, 0.2)),

R> item.id = c(“ITEM1”, “ITEM2”,

R> “ITEM3”, “ITEM4”, “ITEM5”),

R> cats = 2, model = c(“1PLM”,

R> “2PLM”, “3PLM”,

R> “3PLM”, “DRM”))

R> **print**(x)

id cats model par.1 par.2 par.3

1 ITEM1  2  1PLM  1.0  0.2  NA

2 ITEM2  2  2PLM  1.2  2.3  NA

3 ITEM3  2  3PLM  0.5  -0.4  0.2

4 ITEM4  2  3PLM  1.7  -2.5  0.1

5 ITEM5  2  DRM  0.7  0.9  0.2

In this example, the par.drm argument defines the item parameters, where *a* represents the discrimination, *b* indicates the difficulty, and *g* is the guessing parameter (only relevant for models such as the 3PLM and DRM). The item.id argument provides a unique ID for each item, such as “ITEM1”, “ITEM2”, and so on. If item.id=NULL, the item IDs will be automatically assigned. The cats=2 argument specifies that all items are dichotomous, meaning each item has 2 response categories (e.g., correct [1] or incorrect [0]). Finally, the model argument lists the IRT model assigned to each item, including 1PLM, 2PLM, 3PLM, and DRM. If all items in a test follow the same model, such as 3PLM, the model can be specified more conveniently as model=“3PLM”.

## Applying IRT analyses in irtQ

In the previous section, we first demonstrate how to fit various dichotomous IRT models to test data using the est_irt() function, which employs the MMLE-EM algorithm for estimating item parameters. This is followed by a step-by-step illustration of how to estimate examinees’ latent abilities using the est_score() function, which offers several IRT scoring methods. Additionally, the computation of item and test information, as well as characteristic curves, is illustrated. To apply IRT analysis, we explain how to calibrate pretest items using 2 functions: est_irt() and est_item(). The former supports the FIPC method [7], while the latter performs pretest item calibration using the FAPC method [8].

For the illustrations in the previous section, we use a simulated response dataset for a 30-item linear test consisting of binary response patterns from 2,000 examinees. In this section, we employ another simulated dataset that includes 50 operational items and 10 pretest items, where the first 50 items are operational and the remaining 10 are pretest items. In this dataset, all 2,000 examinees responded to the 50 operational items, while each examinee was randomly assigned 5 out of the 10 pretest items. As a result, each pretest item received approximately 1,000 responses, with the remaining responses marked as missing (NAs). Additionally, it is assumed that the 50 operational items have already been calibrated using the IRT 3PLM. Hereafter, the 30-item dataset is referred to as SIMDAT30, and the 60-item dataset with 50 operational and 10 pretest items as SIMDAT60 in the following illustrations. The R code used to generate these simulated datasets is provided in Supplement 1. Note that response data under various IRT models can be simulated using the simdat() function in the irtQ package. For more information on simulating item response data, refer to the irtQ documentation (https://cran.r-project.org/web/packages/irtQ/irtQ.pdf)

### Illustration of item calibration and scoring

We applied dichotomous IRT models, including the 1PLM, 2PLM, and 3PLM, to demonstrate item calibration and scoring, to the SIMDAT30 dataset using the est_irt() function from the irtQ package. The dataset “SIMDAT30.csv” is provided in Supplement 2. At first, the SIMDAT30 dataset can be loaded into R as follows (with “.../” indicating the file path where the dataset is located):

R> simdat30 <- **read.csv**(file = “…/SIMDAT30.csv”,

R> header = FALSE)

Initially, we fit the 1PLM to the SIMDAT30 data. The 1PLM assumes equal item discrimination (*a*) parameters across all items by default. However, if fix.a.1pl=TRUE is specified, a constant value for the *a*-parameters is provided through the a.val.1pl argument, the model fixes the *a*-parameters to that value. Otherwise, the constrained *a*-parameter is estimated automatically. The following R code demonstrates how to fit the 1PL model using the est_irt() function:

R> **library**(irtQ)

R> mod1pl <- **est_irt**(data = simdat30, D = 1.702,

R> model = “1PLM”, cats = 2, fix.a.1pl = FALSE)

Parsing input...

Estimating item parameters...

 EM iteration: 8, Loglike: -29228.5107, Max-Change: 0.00000

Computing item parameter var-covariance matrix...

Estimation is finished in 2.4 seconds.

A string vector indicating the models must be provided in the model argument to specify the IRT models for the data. Available dichotomous IRT models include 1PLM, 2PLM, 3PLM, and DRM (which is treated as 3PLM). For instance, if a test consists of 5 items where the first 3 items use the 1PLM, the fourth item uses the 2PLM, and the fifth item uses the 3PLM, you would specify model=c(“1PLM”, “1PLM”, “1PLM”, “2PLM”, “3PLM”). If a single model is provided (e.g., model=“3PLM”), it will be applied to all items. Additionally, the cats argument requires a vector of unique score category numbers for the items. For example, for a 5-item test where each item has 2 score categories, you would specify cats=c(2, 2, 2, 2, 2). If a single value is provided (e.g., cats=2), it will be applied across all items. The D argument is a scaling constant for logistic IRT models. Setting D=1.702 adjusts the logistic function to approximate the normal ogive function as closely as possible.

Once the item calibration is complete, the summary() function provides a comprehensive overview of the IRT model estimation results. Specifically, the summary includes the item parameter estimates along with their respective standard errors, as shown below:

R> **summary**(mod1pl)

­

Call:

est_irt(data = simdat30, D = 1.702, model = "1PLM", cats = 2)

Summary of the Data

Number of Items: 30

Number of Cases: 2000

­

Summary of Estimation Process

 Maximum number of EM cycles: 500

 Convergence criterion of E-step: 1e-04

 Number of rectangular quadrature points: 49

 Minimum & Maximum quadrature points: -6, 6

 Number of free parameters: 31

 Number of fixed items: 0

 Number of E-step cycles completed: 8

 Maximum parameter change: 0

­

Processing time (in seconds)

 EM algorithm: 0.67

 Standard error computation: 1.54

 Total computation: 2.42

­

Convergence and Stability of Solution

 First-order test: Convergence criteria are satisfied.

 Second-order test: The solution is a possible local maximum.

 Computation of variance-covariance matrix:

 The variance-covariance matrix of item parameter estimates is obtainable.

­

Summary of Estimation Results

 -2loglikelihood: 58437.56

 Akaike Information Criterion (AIC): 58499.56

 Bayesian Information Criterion (BIC): 58673.19

 Item Parameters:

  id  cats  model  par.1  se.1  par.2  se.2  par.3  se.3

1   V1   2   1PLM   0.97   0.02   -1.07   0.07   NA   NA

2   V2   2   1PLM   0.97   NA   -1.07   0.07   NA   NA

3   V3   2   1PLM   0.97   NA   0.17   0.04   NA   NA

4   V4   2   1PLM   0.97   NA   -1.49   0.08   NA   NA

5   V5   2   1PLM   0.97   NA   -1.07   0.07   NA   NA

...

25   V25   2   1PLM   0.97   NA   0.21   0.05   NA   NA

26   V26   2   1PLM   0.97   NA   0.44   0.04   NA   NA

27   V27   2   1PLM   0.97   NA   -0.36   0.05   NA   NA

28   V28   2   1PLM   0.97   NA   0.43   0.04   NA   NA

29   V29   2   1PLM   0.97   NA   0.77   0.04   NA   NA

30   V30   2   1PLM   0.97   NA   0.04   0.05   NA   NA

Group Parameters:

   mu sigma2 sigma

estimates  0    1    1

se      NA   NA   NA

The summary above shows that the *a*-parameter (in par.1 column) was estimated to be 0.97 and constrained to be equal across all items. The getirt() function allows for extracting item parameter estimates and other relevant information (e.g., the variance-covariance matrix of item parameter estimates) from the IRT estimation results by specifying the what argument. For example, setting what=“par.est” retrieves the item parameter estimates, while what=“se.est” returns the standard errors associated with those estimates, as shown:

R> par.est <- **getirt**(x = mod1pl,

R> what = “par.est”)

R> **head**(par.est, 3)

  id  cats  model  par.1  par.2  par.3

1 V1   2  1PLM 0.9656143 -1.0743720   NA

2 V2   2  1PLM 0.9656143 -1.0743720   NA

3 V3   2  1PLM 0.9656143  0.1728018   NA

R> se.est <- **getirt**(x = mod1pl, what = "se.est")

R> **head**(se.est, 3)

  id  cats  model  par.1   par.2  par.3

1 V1   2  1PLM 0.02477692 0.06537754   NA

2 V2   2  1PLM      NA 0.06525617   NA

3 V3   2  1PLM      NA 0.04488698   NA

As shown, the item parameter estimates are returned in the item metadata format described in Section 2, which can be used to estimate the latent abilities of examinees (as explained later in this section). Alternatively, the same information can be accessed directly using the $ operator on the model object:

R> **head**(mod1pl**$**par.est, 3)

  id  cats  model  par.1  par.2  par.3

1 V1   2 1PLM 0.9656143 -1.0743720   NA

2 V2   2 1PLM 0.9656143 -1.0743720   NA

3 V3   2 1PLM 0.9656143  0.1728018   NA

R> **head**(mod1pl**$**se.est, 3)

  id  cats  model  par.1  par.2  par.3

1 V1   2 1PLM 0.02477692 0.06537754   NA

2 V2   2 1PLM   NA 0.06525617   NA

3 V3   2 1PLM   NA 0.04488698   NA

To impose a constraint on the *a*-parameters (e.g., setting *a*=1) across all items in the 1PLM, set both fix.a.1pl=TRUE and a.val.1pl=1, as shown below:

R> mod1pl.const <- est_irt(data = simdat30,

R> D =1.702, model = “1PLM”, cats = 2,

R> fix.a.1pl = TRUE, a.val.1pl = 1)

Parsing input...

Estimating item parameters...

 EM iteration: 3, Loglike: -29268.3748, Max-Change: 0.00000

Computing item parameter var-covariance matrix...

Estimation is finished in 0.9 seconds.

In addition to the 1PLM, other IRT models can be applied to the test data. For instance, the 2PLM can be employed for the first 10 items and the 3PLM for the remaining 20 items, as shown below:

R> model <- **c(rep**(“2PLM”, 10), **rep**(“3PLM”, 20))

R> mod.23pl <- **est_irt**(data = simdat30,

R> D = 1.702, model = model, cats = 2,

R> use.gprior = TRUE, gprior = **list**(

R> dist = beta”, params = c(5, 16)))

Parsing input...

Estimating item parameters...

 EM iteration: 15, Loglike: -28060.7817, Max-Change: 3.1e-05

Computing item parameter var-covariance matrix...

Estimation is finished in 3.51 seconds.

In the example code, a beta prior distribution was applied to estimate the item guessing (*g*) parameters by setting use.gprior=TRUE and gprior=list(dist=“beta”, params=c(5, 16)). The gprior argument requires a list with 2 elements: *dist* (specifying the distribution type) and *params* (providing the corresponding distribution parameters). In addition to the beta distribution, other probability distributions (e.g., log-normal, normal) can also be specified through the gprior argument. Similarly, prior distributions can be applied to the item discrimination and difficulty parameters by setting use.aprior=TRUE and use.bprior=TRUE. The specific distributions for these parameters are selected using the aprior and bprior arguments, respectively. To review the estimation results, use the summary() function:

R> **summary**(mod.23pl)

Call:

est_irt(data = simdat30, D = 1.702, model = model, cats = 2,

     use.gprior = TRUE, gprior = list(dist = "beta", params = c(5, 16)))

­

Summary of the Data

 Number of Items: 30

 Number of Cases: 2000

­

Summary of Estimation Process

 Maximum number of EM cycles: 500

 Convergence criterion of E-step: 1e-04

 Number of rectangular quadrature points: 49

 Minimum & Maximum quadrature points: -6, 6

 Number of free parameters: 80

 Number of fixed items: 0

 Number of E-step cycles completed: 15

 Maximum parameter change: 3.071964e-05

­

Processing time (in seconds)

 EM algorithm: 2.75

 Standard error computation: 2.4

 Total computation: 5.35

­

Convergence and Stability of Solution

 First-order test: Convergence criteria are satisfied.

 Second-order test: The solution is a possible local maximum.

 Computation of variance-covariance matrix:

 The variance-covariance matrix of item parameter estimates is obtainable.

­

Summary of Estimation Results

 -2loglikelihood: 56120.31

 Akaike Information Criterion (AIC): 56280.31

 Bayesian Information Criterion (BIC): 56728.38

 Item Parameters:

    id  cats  model  par.1  se.1  par.2  se.2  par.3  se.3

1   V1   2  2PLM  1.32  0.08  -1.08  0.05   NA  NA

2   V2   2  2PLM  1.63  0.10  -1.01  0.05   NA  NA

3   V3   2  2PLM  0.50  0.04  0.05  0.06   NA  NA

...

28 V28   2  3PLM  1.27  0.11  0.49  0.05  0.10  0.02

29 V29   2  3PLM  1.41  0.31  1.65  0.09  0.26  0.02

30 V30   2  3PLM  1.50  0.13  0.09  0.05  0.11  0.02

 Group Parameters:

      mu sigma2 sigma

estimates  0   1   1

se    NA   NA   NA

If needed, the *g*-parameters can be fixed to a specific value (e.g., 0.2) rather than estimated when fitting the 3PLM. To do this, set fix.g=TRUE and provide the desired value using the g.val argument. Additionally, since est_irt() uses the EM algorithm for item parameter estimation, the tolerance criterion for the E-step (e.g., 0.001) and the maximum number of EM cycles (e.g., 200) can be adjusted. The default values are 0.0001 and 500, respectively. The following example illustrates this:

R> mod.23pl.fixg <- **est_irt**(data = simdat30,

R> D = 1.702, model = model, cats = 2,

R> g.val = 0.2, fix.g = TRUE, Etol = 0.001,

R> MaxE = 200)

­

Parsing input...

Estimating item parameters...

 EM iteration: 14, Loglike: -28213.0853, Max-Change: 0.000529

## Computing item parameter var-covariance matrix...

## Estimation is finished in 4.03 seconds.

­

R> **summary**(mod.23pl.fixg)

Call:

est_irt(data = simdat30, D = 1.702, model = model, cats = 2,

      fix.g = TRUE, g.val = 0.2, Etol = 0.001, MaxE = 200)

­

Summary of the Data

 Number of Items: 30

 Number of Cases: 2000

­

Summary of Estimation Process

 Maximum number of EM cycles: 200

 Convergence criterion of E-step: 0.001

 Number of rectangular quadrature points: 49

 Minimum & Maximum quadrature points: -6, 6

 Number of free parameters: 60

 Number of fixed items: 0

 Number of E-step cycles completed: 14

 Maximum parameter change: 0.0005289198

­

Processing time (in seconds)

 EM algorithm: 1.65

 Standard error computation: 2

 Total computation: 4.03

­

Convergence and Stability of Solution

 First-order test: Convergence criteria are satisfied.

 Second-order test: The solution is a possible local maximum.

 Computation of variance-covariance matrix:

 The variance-covariance matrix of item parameter estimates is obtainable.

­

Summary of Estimation Results

 -2loglikelihood: 56424.82

 Akaike Information Criterion (AIC): 56544.82

 Bayesian Information Criterion (BIC): 56880.87

 Item Parameters:

    id  cats  model  par.1  se.1  par.2  se.2  par.3  se.3

1   V1   2  2PLM  1.32  0.08  -1.08  0.05   NA  NA

2   V2   2  2PLM  1.62  0.10  -1.01  0.05   NA  NA

3   V3   2  2PLM  0.49  0.04  0.05  0.06   NA  NA

...

10 V10   2  2PLM  0.96  0.06  -0.58  0.05   NA  NA

11 V11   2  3PLM  1.18  0.10  -1.69  0.10   0.2  NA

12 V12   2  3PLM  1.42  0.10  0.05  0.04   0.2  NA

...

28 V28   2  3PLM  1.58  0.14  0.64  0.04  0.2  NA

29 V29   2  3PLM  0.83  0.10  1.64  0.11   0.2  NA

30 V30   2  3PLM  1.80  0.14  0.22  0.04  0.2  NA

 Group Parameters:

mu sigma2 sigma

estimates  0   1   1

se    NA   NA   NA

The summary shows that the *g*-parameters (in par.3 column) for the last 20 items (Items 11 to 30) were fixed at 0.2 and have no corresponding standard errors since they were not estimated.

By default, the est_irt() function does not estimate the weights of the latent ability distribution and instead fixes them to the standard normal distribution, *N*(0, 1). However, setting EmpHist=TRUE allows the weights to be estimated at the specified quadrature points, which can be controlled via the Quadrature argument. The Quadrature argument takes a numeric vector with 2 components: the number of quadrature points and the symmetric minimum and maximum values for those points. For example, Quadrature=c(49, 6) sets 49 quadrature points ranging from -6 to 6 (the default is c(49, 6)). For more details on estimating weights using the empirical histogram method, refer to Woods [9].

R> mod.emp <- **est_irt**(data = simdat30, D = 1.702,

R> model = model, cats = 2, g.val = 0.2,

R> fix.g = TRUE, Etol = 0.001, MaxE = 200,

R> Quadrature = c(49, 6), EmpHist = TRUE)

Parsing input...

Estimating item parameters...

 EM iteration: 178, Loglike: -28187.3630, Max-Change: 0.00100

Computing item parameter var-covariance matrix...

Estimation is finished in 11.17 seconds.

You can retrieve the estimated weights with the following command:

R> **getirt**(mod.emp, what = "weights")

  theta   weight

1 -6.00 5.005072e-24

2 -5.75 5.005072e-24

3 -5.50 5.005072e-24

...

49 6.00 1.683412e-24

To fit the 3PLM to the SIMDAT30 data with a beta prior distribution for the *g*-parameters, you can use the following code:

R> mod3pl < - **est_irt**(data = simdat30, D = 1.702,

R> model = “3PLM”, cats = 2, use.gprior = TRUE,

R> gprior = list(dist = “beta”, params = c(5,

R> 16)))

Parsing input...

Estimating item parameters...

 EM iteration: 50, Loglike: -27995.2943, Max-Change: 9.6e-05

Computing item parameter var-covariance matrix...

Estimation is finished in 6.05 seconds.

R> **getirt**(mod3pl, what = "par.est")

  id  cats  model  par.1  par.2  par.3

1  V1  2  3PLM 1.386443 -1.01413626 0.12159492

2  V2  2  3PLM 1.735638 -0.96314036 0.10642896

3  V3  2  3PLM 1.101382  0.70970908 0.28508382

...

30 V30  2  3PLM 1.519163  0.05547073 0.10834886

Once the item parameters are estimated using the specified IRT models, the latent ability parameters of individuals can be estimated using the est_score() function. The ability parameters are estimated using the method specified in the method argument by passing the est_irt object (from the est_irt() function) to the x argument. The est_score() function supports various IRT scoring methods, including maximum likelihood estimation (ML), maximum likelihood estimation with fences (MLF) [10], weighted likelihood estimation (WL) [11], maximum a posteriori estimation (MAP) [12], expected a posteriori estimation (EAP) [13], EAP summed scoring (EAP.SUM) [14,15], and inverse test characteristic curve scoring (INV.TCC) [16]. The following code demonstrates the ability estimation using the EAP scoring method and the 3PLM calibration results from the previous example:

R> score.eap <- **est_score**(x = mod3pl,

R> method = “EAP”, norm.prior = c(0, 1),

R> nquad = 41)

Since EAP (and MAP) are Bayesian estimators, a prior distribution for latent abilities must be specified. In est_score(), the default prior is a normal distribution, which can be modified using the norm.prior argument (mean and standard deviation). The nquad argument specifies the number of Gaussian quadrature points for the normal prior distribution.

Alternatively, the same scoring results can be obtained without using the est_irt object. By providing the item metadata (containing item parameter estimates from item calibration) in the x argument, and the item response data set in the data argument, est_score() can compute the ability estimates. The example below demonstrates this approach using the same 3PLM calibration results:

R> score.eap <- **est_score**(x = par.3pl, data =

R> simdat30, D = 1.702, method = “EAP”,

R> norm.prior = c(0, 1), nquad = 41)

R> **head**(score.eap, 5)

   est.theta  se.theta

1  0.4062983 0.2201802

2 -0.8969595 0.2772995

3  0.5426724 0.2211188

4 -1.2891101 0.3259248

5  0.7060294 0.2571311

As shown above, est_score() returns a data frame with 2 columns: the ability estimates (1st column) and their corresponding standard errors (2nd column). In addition to the EAP method, you can choose from various IRT scoring methods (e.g., ML, WL, MAP) via the method argument. For example, the ML method can be applied with the range argument to limit the ability estimates within a specified range (e.g., between -5 and 5).

R> score.ml <- **est_score**(x = par.3pl,

R> data = simdat30, D = 1.702,

R> method = “ML”, range = c(-5, 5))

R> **head**(x = score.ml, 5)

   est.theta  se.theta

1  0.4111583 0.2354412

2 -0.8839326 0.2728941

3  0.5890208 0.2406527

4 -1.3502772 0.3868866

5  0.7462858 0.2453933

Using the item parameter estimates from est_irt(), both the item and test characteristic curves (ICC and TCC) and the item and test information functions (IIF and TIF) can be computed with the traceline() and info() functions. For both functions, the item metadata should be provided in the x argument, and a vector of discrete ability values should be passed to the theta argument, where the characteristic and information function values are calculated. The following code demonstrates computing ICC/TCC and IIF/TIF for theta values ranging from -4 to 4 in increments of 0.1, using the 3PLM calibration results:

R> trace.3pl <- **traceline**(x = par.3pl,

R> theta = theta, D = 1.702)

R> info.3pl <- **info**(x = par.3pl, theta = theta,

R> D = 1.702)

**Fig. 1.**
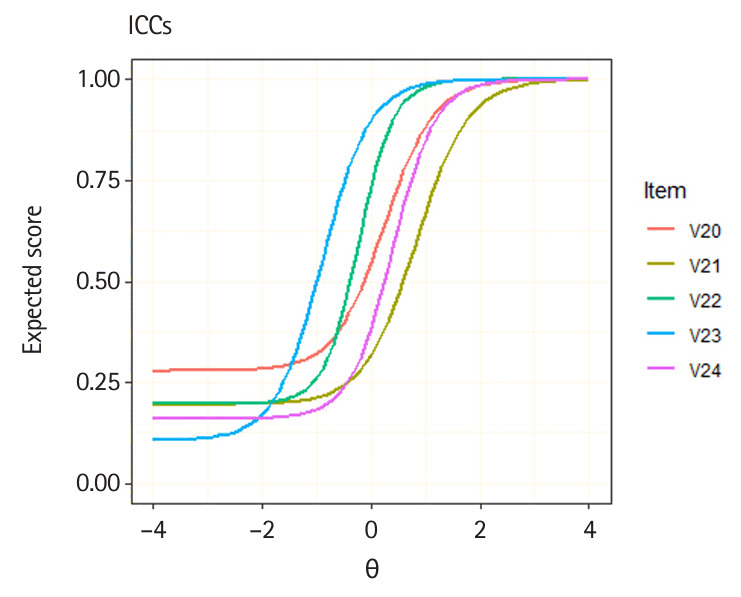
Illustrations of item characteristic curves (ICCs) for the 5 dichotomous items.

**Fig. 2.**
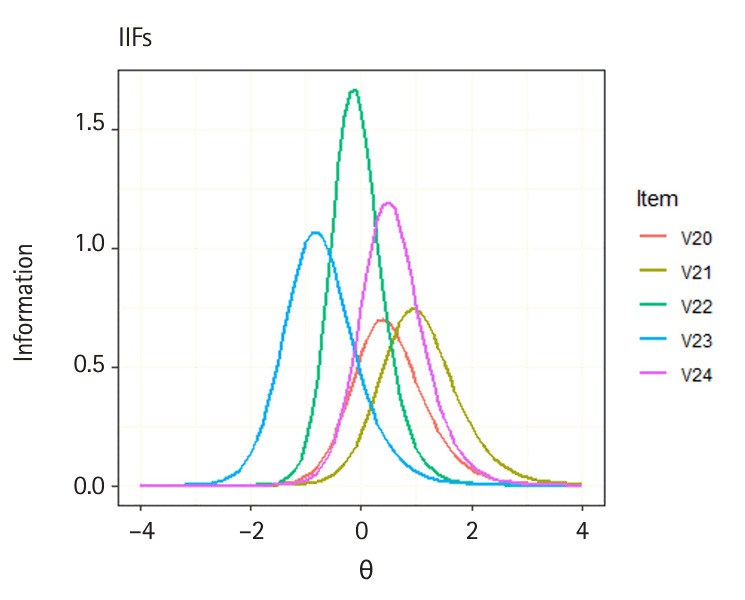
Illustrations of item information functions (IIFs) for the 5 dichotomous items.

The plot() method can be used to draw ICC for each score category and IIF using objects of class traceline and info provided in the x argument. The following sample code demonstrates how to generate ICC and IIF plots for a specific item. In this example, ICCs and IIFs for multiple items are plotted by providing a vector of item locations in the item.loc argument. To display only the expected score curve for multiple ICCs, set score.curve=TRUE in the plot() function. Additionally, multiple ICCs or IIFs can be overlapped in the same panel by setting overlap=TRUE. The following sample code demonstrates how to create ICC and IIF plots for 5 items (Figs. 1, 2).

R> **plot**(x = trace.3pl, item.loc = 20:24,

R> score.curve = TRUE, overlap = TRUE,

R> main.text = “ICCs”)

R> **plot**(x = info.3pl, item.loc = 20:24,

R> overlap = TRUE, main.text = “IIFs”)

**Fig. 3.**
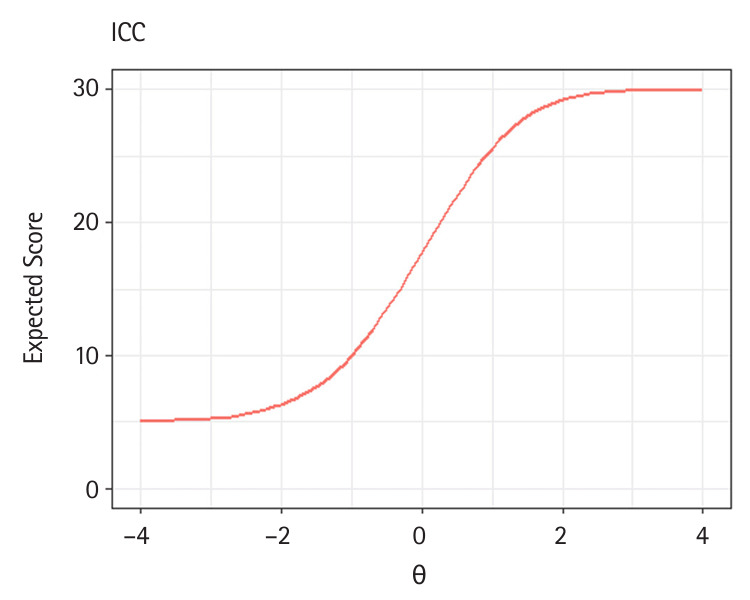
Illustrations of test characteristic curve (TCC) of the 30-item test.

**Fig. 4.**
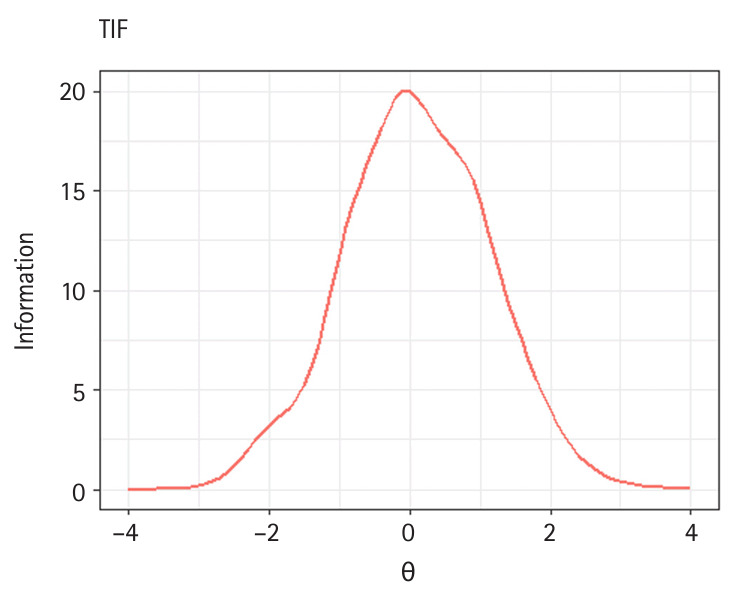
Illustrations of test information function (TIF) of the 30-item test.

Also, when item.loc=NULL in the plot() method, either the TCC or TIF is generated, depending on the type of object provided. The following code demonstrates how to produce TCC and TIF plots (Figs. 3, 4).

R> **plot**(x = trace.3pl, item.loc = NULL,

R> main.text = “TCC”)

R> **plot**(x = info.3pl, item.loc = NULL,

R> main.text = “TIF”)

## Illustration of estimating pretest item parameters

We illustrate how to estimate pretest item parameters using the SIMDAT60 dataset with both FIPC and FAPC methods. FIPC is widely used for calibrating pretest items by placing their parameters on the same scale as operational items, without the need for post-hoc linking or scaling [17], especially in CAT environments. In FIPC, the operational item parameters are fixed, while the underlying latent ability distribution is estimated during the calibration process to align pretest item parameters with the operational scale [7]. FAPC, also known as Stocking’s Method A [11], is a simpler and more direct approach to pretest item calibration. It estimates item parameters by maximizing the likelihood given the examinees’ ability estimates. FAPC is known to produce accurate and unbiased item parameter estimates, especially when pretest items are administered randomly, as opposed to adaptively [17,18].

In the irtQ package, FIPC is implemented using the est_irt() function, where pretest item parameters are estimated while the operational item parameters are fixed. In the SIMDAT60 dataset, the first 50 items are operational, and the remaining 10 are pretest items. The 10 pretest items are estimated during calibration, while the parameters for the 50 operational items remain fixed.

Before calibrating pretest items with FIPC, an item metadata set must be prepared and provided in the x argument of the est_irt() function. This metadata set should include the fixed operational item parameters and placeholders (e.g., 0 or NA) for estimating the pretest item parameters. It is important to correctly specify the unique score category numbers and the fitted IRT models for all items in the second and third columns of the metadata. Supplement 1 in the supplementary materials provides the R code for generating the item metadata, which includes both operational and pretest items, using the shape_df() function.

The first and last 10 items from the item metadata used in the pretest calibration illustration are shown below. The complete item metadata, ‘ITEMMETA_EX2.csv’, is available in Supplement 2 of the supplementary materials. Placeholders for the last 10 pretest items (Items 51 to 60) are set as 1 for the *a*-parameters, 0 for the *b*-parameters, and 0.2 for the *g*-parameters.

R> (x <- **read.csv**(file =

R> **file.path**(“.../ITEMMETA_EX2.csv”),

R> header =TRUE))

  id  cats  model  par.1  par.2  par.3

1  OP1  2  3PLM  1.66  -1.24  0.09

2  OP2  2  3PLM  1.56  -0.46  0.16

3  OP3  2  3PLM  1.09  -0.83  0.17

4  OP4  2  3PLM  1.38  0.34  0.02

5  OP5  2  3PLM  1.80  1.07  0.15

6  OP6  2  3PLM  1.85  1.22  0.21

7  OP7  2  3PLM  1.15  0.74  0.03

8  OP8  2  3PLM  1.48  -0.48  0.27

9  OP9  2  3PLM  1.01  0.56  0.22

10  OP10  2  3PLM  1.53  -1.25  0.22

...

51  PT1  2  3PLM  1.00  0.00  0.20

52  PT2  2  3PLM  1.00  0.00  0.20

53  PT3  2  3PLM  1.00  0.00  0.20

54  PT4  2  3PLM  1.00  0.00  0.20

55  PT5  2  3PLM  1.00  0.00  0.20

56  PT6  2  3PLM  1.00  0.00  0.20

57  PT7  2  3PLM  1.00  0.00  0.20

58  PT8  2  3PLM  1.00  0.00  0.20

59  PT9  2  3PLM  1.00  0.00  0.20

60  PT10  2  3PLM  1.00  0.00  0.20

Next, the SIMDAT60 dataset needs to be loaded into R as follows:

R> simdat60 <- read.csv(file =

R> file.path(“.../SIMDAT60.csv”),

R> header = FALSE)

R> **tail**(simdat60[, 50:60], n = 8)

   V51 V52 V53 V54 V55 V56 V57 V58 V59 V60

1993  NA  0  1  NA  NA  NA  1  NA  0  0

1994  NA  0  0  NA  0  NA  0  NA  0  NA

1995  1  0  1  NA  0  NA  NA  NA  0  NA

1996  1  0  NA  NA  1  0  0  NA  NA  NA

1997  1  NA  NA  NA  NA  0  NA  0  0  0

1998  1  0  NA  1  NA  NA  0  NA  0  NA

1999  1  0  1  NA  NA  1  NA  1  NA  NA

2000  1  NA  1  1  NA  NA  NA  0  NA  1

The SIMDAT60 dataset contains missing values (NAs) in the columns corresponding to pretest items. To run FIPC with this data, set fipc=TRUE in the est_irt() function. Next, choose a specific FIPC method using the fipc.method argument, such as fipc.method=“MEM”. The available methods are: (1) OEM: no prior weights updating and one EM cycle (NWU-OEM) [19]. This method uses a single E-step based on fixed items and a single M-step for non-fixed items. (2) MEM: multiple prior weights updating and multiple EM cycles (MWU-MEM) [7]. This method iteratively updates the latent ability distribution and non-fixed item parameters. In the first EM cycle, the process is the same as NWU-OEM, but from the second cycle onwards, both the parameters of non-fixed items and the prior weights are updated concurrently.

For more details about the FIPC procedure, refer to Kim [7]. Additionally, a vector of integer values specifying the location of the operational items to be fixed should be provided in the fix.loc argument (e.g., fix.loc=1:50). Since the SIMDAT60 dataset contains missing values, the missing argument must be used to indicate the missing value representation (e.g., missing=NA). The following code illustrates how to implement FIPC for the SIMDAT60 dataset:

R> mod.fipc < - **est_irt**(x = x, data = simdat60,

R> D = 1.702, use.gprior = TRUE,

R> gprior = **list**(dist = “beta”, params = c(5,

R> 16)), missing = NA, Etol = 0.001,

R> MaxE = 500, fipc = TRUE,

R> fipc.method = “MEM”, fix.loc = 1:50)

Parsing input...

Estimating item parameters...

 EM iteration: 7, Loglike: -49085.4942, Max-Change: 0.000987

Computing item parameter var-covariance matrix...

Estimation is finished in 3.38 seconds.

To review the estimation results, use the summary() function as below:

R> **summary**(mod.fipc)

Call:

est_irt(x = x, data = simdat60, D = 1.702, use.gprior = TRUE,

   gprior = list(dist = "beta", params = c(5, 16)), Etol = 0.001,

   MaxE = 500, fipc = TRUE, fipc.method = "MEM", fix.loc = 1:50)

­

Summary of the Data

 Number of Items: 60

 Number of Cases: 2000

­

Summary of Estimation Process

 Maximum number of EM cycles: 500

 Convergence criterion of E-step: 0.001

 Number of rectangular quadrature points: 49

 Minimum & Maximum quadrature points: -6, 6

 Number of free parameters: 32

 Number of fixed items: 50

 Number of E-step cycles completed: 7

 Maximum parameter change: 0.0009871109

­

Processing time (in seconds)

 EM algorithm: 0.8

 Standard error computation: 1.39

 Total computation: 3.38

­

Convergence and Stability of Solution

 First-order test: Convergence criteria are satisfied.

 Second-order test: The solution is a possible local maximum.

 Computation of variance-covariance matrix:

   The variance-covariance matrix of item parameter estimates is obtainable.

­

Summary of Estimation Results

 -2loglikelihood: 98170.99

 Akaike Information Criterion (AIC): 98234.99

 Bayesian Information Criterion (BIC): 98414.22

 Item Parameters:

    id  cats  model  par.1  se.1  par.2  se.2  par.3  se.3

1   OP1   2  3PLM  1.66  NA  -1.24  NA  0.09  NA

2   OP2   2  3PLM  1.56  NA  -0.46  NA  0.16  NA

3   OP3   2  3PLM  1.09  NA  -0.83  NA  0.17  NA

...

50  OP50   2  3PLM  2.01  NA  1.13  NA  0.26  NA

51  PT1   2  3PLM  1.34  0.17  -1.46  0.15  0.21  0.08

52  PT2   2  3PLM  1.08  0.22  1.07  0.10  0.26  0.03

53  PT3   2  3PLM  2.16  0.25  -0.47  0.06  0.17  0.04

54  PT4   2  3PLM  2.38  0.37  -0.43  0.06  0.24  0.04

55  PT5   2  3PLM  2.12  0.29  0.95  0.05  0.17  0.02

56  PT6   2  3PLM  2.24  0.23  0.05  0.04  0.13  0.02

57  PT7   2  3PLM  1.10  0.19  0.96  0.10  0.25  0.03

58  PT8   2  3PLM  2.11  0.23  0.84  0.04  0.03  0.01

59  PT9   2  3PLM  1.46  0.24  1.29  0.07  0.13  0.02

60  PT10   2  3PLM  1.83  0.21  1.02  0.04  0.06  0.01

 Group Parameters:

   mu sigma2 sigma

estimates  0.02  1.06  1.03

se     0.02   0.03  0.02

From the summary of FIPC calibration, it is evident that only the pretest item parameters (Items 51 to 60) were estimated, with corresponding standard errors. Operational item parameters were fixed during FIPC, so their standard errors were not computed. Additionally, the mean (mu), variance (sigma2), and standard deviation (sigma) of the latent ability distribution were estimated, as shown under “Group Parameters.” Since the true ability parameters for 2,000 examinees were randomly sampled from *N*(0, 1) when generating the SIMDAT60 data (Supplement 1), the estimated mean and standard deviation quite accurately reflect the true N(0, 1) distribution.

When EmpHist=TRUE, both the scale (mean and variance) and the weights of the latent ability distribution are estimated. If EmpHist=FALSE, a normal distribution is used for the latent ability distribution, and only its scale is updated during the EM cycle, while the weights for the quadrature points are derived from the normal distribution. The following code illustrates the FIPC process by estimating both the scale and weights of the latent ability distribution:

R> mod.fipc.emp <- **est_irt**(x = x,

R> data = simdat60, D = 1.702,

R> use.gprior = TRUE,

R> gprior = **list**(dist =

R> “beta”, params = c(5, 16)),

R> EmpHist = TRUE,

R> Etol = 0.001, MaxE = 500,

R> fipc = TRUE,

R> fipc.method = “MEM”,

R> fix.loc = 1:50)

Parsing input...

Estimating item parameters...

 EM iteration: 9, Loglike: -49079.5704, Max-Change: 0.000652

Computing item parameter var-covariance matrix...

Estimation is finished in 1.51 seconds.

R> **getirt**(mod.fipc.emp, what = "weights")

   theta  weight

1  -6.00 1.412474e-09

2  -5.75 6.132156e-09

3  -5.50 2.500244e-08

...

47  5.50 5.165166e-08

48  5.75 1.270338e-08

49  6.00 2.931665e-09

Next, we demonstrate how to use the FAPC method to calibrate pretest items by applying the est_item() function with the SIMDAT60 dataset. The use of est_item() is straightforward, as it shares many of the same arguments as the est_irt() function. However, a key difference is that we must provide a vector of ability parameters through the score argument since est_item() estimates pretest item parameters based on these fixed latent ability estimates. In practice, ability parameters are typically estimated using responses from operational items only. For illustration, we first estimate the ability parameters of 2,000 examinees using the responses to the 50 operational items in the SIMDAT60 dataset. The ML scoring method is used in this example:

R> x_op <- x[1:50, ]

R> simdat60_op <- simdat60[, 1:50]

R> score_op <- **est_score**(x = x_op,

R> data = simdat60_op, D = 1.702,

R> method = “ML”, range = c(-5, 5))

Ability estimates need to be extracted from the score_op object obtained from the est_score() function, and a response data matrix containing only the pretest items should be prepared to execute the FAPC method. The following code demonstrates the FAPC process for the 10 pretest items in the SIMDAT60 dataset. A vector of item IDs for the pretest items is provided via the item.id argument to assign a unique ID to each item.

R> mod.fapc < - **est_item**(data = simdat60_pt,

R> score = est.th, D = 1.702, model = “3PLM”,

R> cats = 2, item.id = paste0(“PT”, 1:10),

R> use.gprior = TRUE,

R> gprior = list(dist = “beta”,

R> params = c(5, 16)), missing = NA)

Starting...

Parsing input...

Estimating item parameters...

Estimation is finished.

To review the estimation results of the FAPC method, run the summary() function:

R> **summary**(mod.fapc)

Call:

est_item(data = simdat60_pt, score = est.th, D = 1.702, model = "3PLM",

   cats = 2, item.id = paste0("PT", 1:10), use.gprior = TRUE,

   gprior = list(dist = "beta", params = c(5, 16)), missing = NA)

­

Summary of the Data

 Number of Items in Response Data: 10

 Number of Excluded Items: 0

 Number of free parameters: 30

 Number of Responses for Each Item:

    id   n

1  PT1  991

2  PT2  966

3  PT3  951

4  PT4  1002

5  PT5  1007

6  PT6  1049

7  PT7  977

8  PT8  1001

9  PT9  1005

10  PT10  1051

­

Processing time (in seconds)

 Total computation: 1.46

­

Convergence of Solution

 All item parameters were successfully converged.

­

Summary of Estimation Results

 -2loglikelihood: 8477.85

 Item Parameters:

    id  cats  model  par.1  se.1  par.2  se.2  par.3  se.3

1  PT1   2  3PLM  1.23  0.16  -1.50  0.17  0.25  0.08

2  PT2   2  3PLM  0.80  0.15  1.11  0.13  0.23  0.04

3  PT3   2  3PLM  1.91  0.20  -0.48  0.06  0.17  0.03

4  PT4   2  3PLM  2.07  0.24  -0.44  0.07  0.24  0.04

5  PT5   2  3PLM  1.64  0.19  0.98  0.05  0.16  0.02

6  PT6   2  3PLM  1.98  0.18  0.05  0.04  0.13  0.02

7  PT7   2  3PLM  0.82  0.14  0.99  0.12  0.22  0.04

8  PT8   2  3PLM  1.85  0.16  0.89  0.04  0.03  0.01

9  PT9   2  3PLM  1.17  0.18  1.38  0.08  0.12  0.02

10  PT10   2  3PLM  1.57  0.15  1.07  0.05  0.05  0.01

Group Parameters:

   mu sigma

0.03  1.23

## Limitations

This study only covers some details of IRT model fitting and analysis procedures available in the irtQ package. Users are encouraged to refer to the official irtQ manual for more comprehensive guidance. Due to space limitations, we could not address several valuable functions within the package, such as:

• est_mg(): To conduct multiple-group item calibration using the MMLE-EM algorithm [20].

• irtfit() and sx2_fit(): To evaluate model-data fit at the item level using the traditional item fit statistics (e.g., *χ*^2^ fit statistics) and the S-X2 statistic [21]

• bring.flexmirt(), bring.bilog(), bring.parscale(), bring.mirt(): To import item and ability parameters from various IRT software, including flexMIRT, Bilog-MG3, PARSCALE, and the mirt R package.

• lwrc(): To compute the conditional probability distribution of observed number-correct scores using Lord and Wingersky’s recursive formula [22].

• cac_lee() and cac_rud(): To calculate classification accuracy and consistency indices using methods proposed by Lee [23] and Rudner [24,25].

Additionally, several functions are specifically designed for CAT environments, including:

• rdif() and catsib(): To detect differential item functioning (DIF) using the residual-based DIF detection framework [26,27] and the CATSIB method [28].

• reval_mst(): To evaluate the measurement precision of a multistage-adaptive test panel using a recursion-based evaluation method [29].

• pcd2(): To calculate the pseudo-count D2 statistic [30] to evaluate item parameter drift in CAT.

Despite its broad functionality, the irtQ package is still under active development. With its ongoing improvements, the irtQ package promises to become an increasingly valuable tool for researchers and practitioners working within the IRT framework, offering practical and efficient solutions for a wide range of psychometric applications.

## Conclusion

In this paper, we presented the key features of the irtQ package and demonstrated its use for parameter estimation, ability scoring, and other essential IRT analyses. Specifically, we highlighted how the est_irt() function can be applied to estimate item parameters, including calibrating pretest items using the MMLE-EM algorithm. We also provided a step-by-step guide for estimating latent abilities using the est_score() function, as well as illustrating how to compute and visualize ICCs/TCCs and IIFs/TIFs.

## Figures and Tables

**Figure f5-jeehp-21-23:**
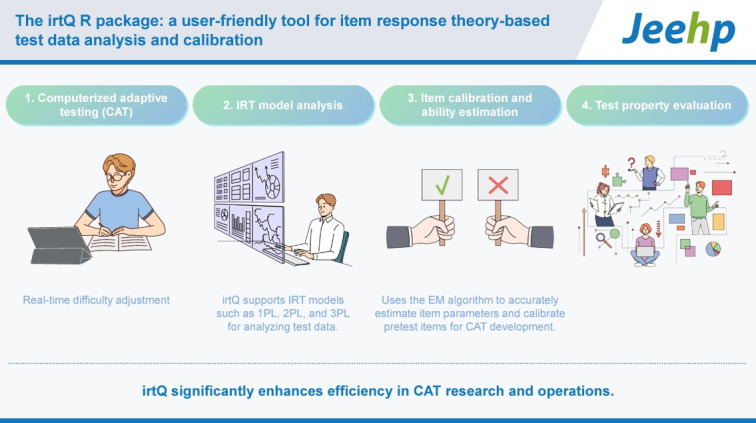


**Table 1.** The illustrative item metadata for 5 dichotomous item response theory items

id	cats	model	par.1	par.2	par.3
ITEM1	2	1PLM	1	0.2	NA
ITEM2	2	1PLM	1.2	2.3	NA
ITEM3	2	2PLM	0.5	-0.4	0.2
ITEM4	2	3PLM	1.7	-2.5	0.1
ITEM5	2	DRM	0.7	0.9	0.2

1PLM, 1-parameter logistic model; 2PLM, 2-parameter logistic model; 3PLM, 3-parameter logistic model; DRM, dichotomous response model.

## Supplementary materials

Supplementary files are available from Harvard Dataverse: https://doi.org/10.7910/DVN/W8PPDH

Supplement 1. A sample R script used to generate 2 simulated datasets (SIMDAT30 and SIMDAT60).

Supplement 2. A zipped file containing the 2 simulated datasets (‘SIMDAT30.csv’ and ‘SIMDAT60.csv’) along with the item metadata file (‘ITEMMETA_EX2.csv’).

Supplement 3. Audio recording of the abstract.
